# Supplementary material for: Reward type influences adults’ rejections of inequality in a task designed for children
Source: PLoS One. 2022 Aug 16;17(8):e0272710. doi: 10.1371/journal.pone.0272710 (PMC9380955; doi:10.1371/journal.pone.0272710)
Supplement: S1 File — (PDF) [file pone.0272710.s001.pdf]

Supplemental Materials for *Reward type influences adults' rejections of inequality in a task designed for children*

Table of Contents

Table S1: Sample breakdown by condition, reward type and actor gender 2

Random subset analyses 3

Table S2: Cells sizes post random sampling 3

Figure S1. Results from random subset 4

Table S3. Estimate and bootstrapped CIs... 5

Figure S2: Relationship between rejections and age 6

Table S4: Output from sensitivity analyses.. 7

Trial analyses 8

Figure S3. Rejections across trials (trials 1-12) 8

Figure S4. Rejections across trials by distribution (trials 1-6).. 9

Table S5. Estimate and bootstrapped CIs... 10

Table S1: Sample breakdown by condition, reward type and actor gender

		Female	Male	Total
Disadvantageous	Skittles	21	11	**32**
	Token	10	12	**22**
Advantageous	Skittles	21	9	**30**
	Token	11	9	**20**

Randomly sampling data to match reward type by condition cell sizes and associated analyses

Rationale

Our sample of participants was imbalanced with respect to the number of people who made decisions about tokens versus the number who made decisions about Skittles. This imbalance was due to the fact that we originally planned to exclude participants who did not like Skittles and thus tested additional pairs to replace pairs in which actors did not like Skittles. However, we later realized that this would lead to an asymmetric exclusion criteria across reward type because we did not have the equivalent criterion for the token reward type. Consequently, we chose not to exclude those participants. We address participants' valuation of reward types in the Discussion section of our main text.

To address the imbalanced sample with respect to reward type, we created an additional data set that removed a random subset of participants tested with Skittles to match the sample of participants tested with Tokens (this subset excluded a random subset of 10 participants in the advantageous condition and 10 participants in the disadvantageous condition). We then confirmed that our main model was robust to the exclusion of this subset (GLMM, 3-way interaction between condition, distribution and resource type: LRT, $\chi^2_1 = 5.83$, $p = 0.016$).

Table S2: Cells sizes post random sampling

		Total	Subset total
Disadvantageous	Skittles	**32**	**22**
	Token	**22**	**22**
Advantageous	Skittles	**30**	**20**
	Token	**20**	**20**

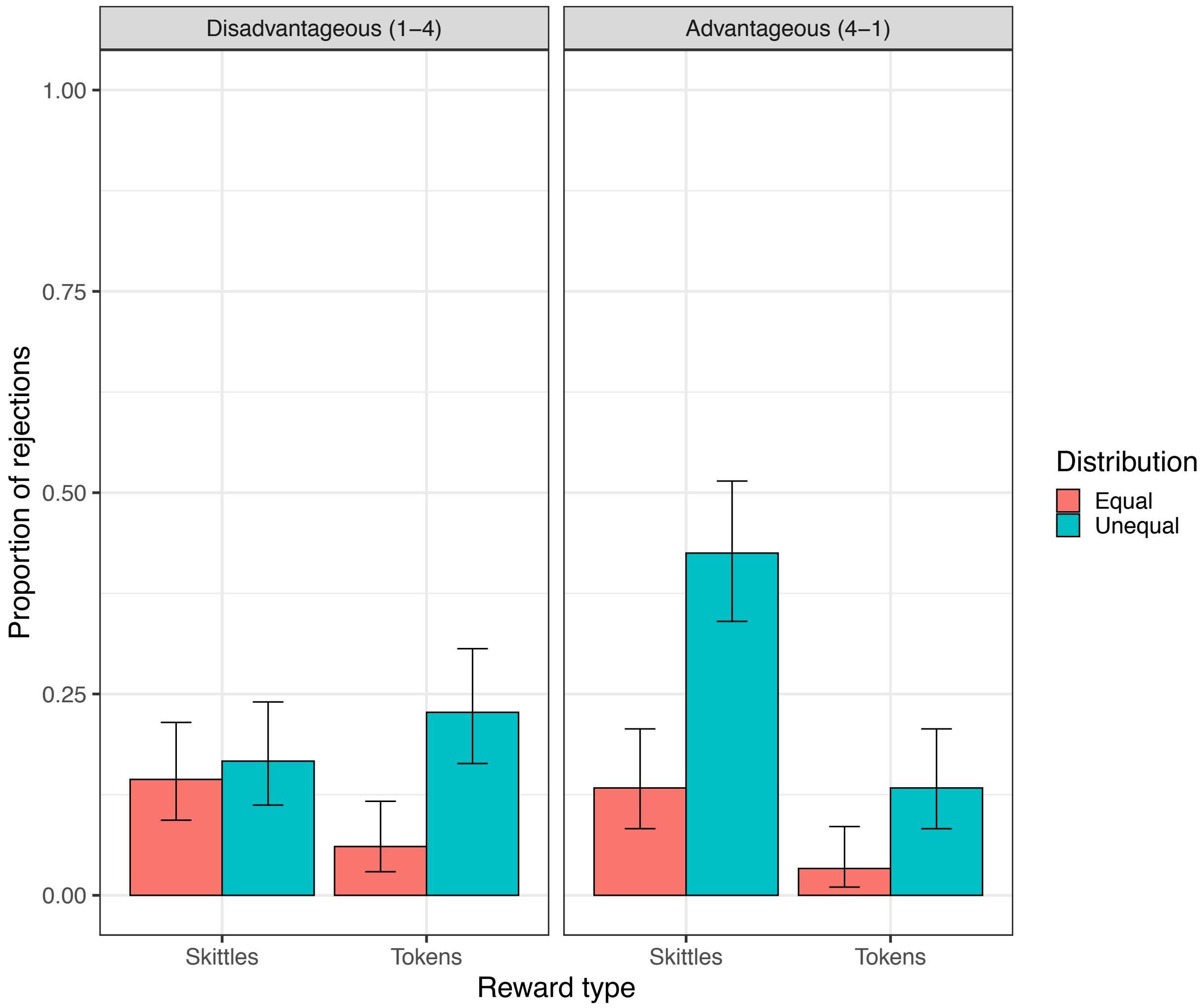

Figure S1. Results from random subset showing proportion of rejections of equal and unequal allocations in disadvantageous and advantageous conditions. Rejections are shown by reward type. Error bars show binomial confidence intervals.

Table S3. Estimates and bootstrapped CIs (*N* = 1,000 simulations) of fixed effects in Generalized Linear Mixed Model predicting children's rejections (= 1) in the Inequity Game. This model was run on the random subset that matched cell sizes by reward type within condition. Baselines were set as follow: Condition: AI; Distribution = Equal; Resource type = Skittles. Table also shows goodness-of-fit statistics.

	**Full model with random subset**
(Intercept)	-3.36*
	[-5.04; -2.16]
Condition: DI	0.41
	[-1.69; 2.31]
Distribution: Unequal	2.61*
	[1.68; 3.81]
Resource type: Tokens	-1.95
	[-8.82; 0.08]
Condition x Distribution	-2.38*
	[-3.84; -1.12]
Condition x Resource type	0.17
	[-3.24; 6.88]
Distribution x Resource type	-0.64
	[-2.25; 5.17]
Condition x Distribution x Resource type	2.57
	[-3.32; 5.18]
AIC	673.93
BIC	718.17
Log Likelihood	-327.97
Number of trials	1008
Number of participants	84
Variance: ID (Intercept)	5.51

* 0 outside the confidence interval

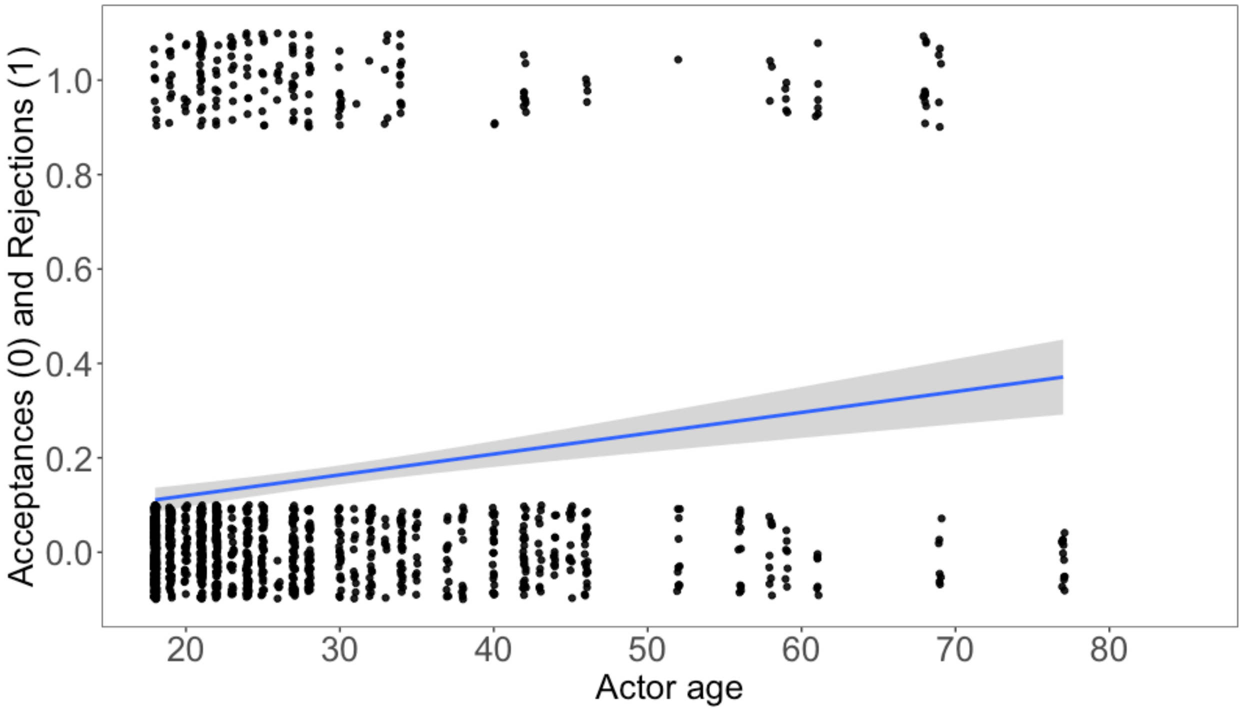

Figure S2. Relationship between rejections and age

Table S4. Output from sensitivity analysis using 'simr'. We first narrowed in on a range of plausible effects by running a series of preliminary simulations. Based on these simulations we focused in on a range of effects to test -3 to 3). We ran simulations in these ranges (N = 11), stepping between effects by 0.5.

Main model: 3-way interaction (Condition x Distribution x Reward type)		DI model: two-way interaction (Distribution x Reward type)		AI model: two-way interaction (Distribution x Reward type)	
-3	0.15	-3	0.2	-3	0.72
-2.5	0.2	-2.5	0.29	-2.5	0.68
-2	0.17	-2	0.25	-2	0.58
-1.5	0.14	-1.5	0.26	-1.5	0.43
-1	0.1	-1	0.19	-1	0.25
-0.5	0.02	-0.5	0.12	-0.5	0.08
0	0.01	0	0.02	0	0.03
0.5	0.02	0.5	0.05	0.5	0.05
1	0.1	1	0.27	1	0.21
1.5	0.25	1.5	0.66	1.5	0.34
2	0.37	2	0.91	2	0.56
2.5	0.53	2.5	0.98	2.5	0.72
3	0.71	3	0.98	3	0.86

Trial analyses

In a set of exploratory analyses, we examined whether actors' decisions varied across trials. We did this in two ways. First, we included trial number (1-12) as a predictor. Second, we included trial by distribution (trials 1-6 of equal allocation and trials 1-6 of unequal allocations) as a predictor. In our models examining the effect of trial by distribution (1-6 of equal and unequal trials), we examine the interaction between trial (1-6) and distribution (equal and unequal). We have plotted these effects by condition (advantageous and disadvantageous) and resource types (see Figs. S3 and S4).

After initially running these models on the full data set, we then conducted these two categories of trial analysis separately for the advantageous and disadvantageous conditions to mirror the analytical approach we present in the main text. As Table S5 shows, these analyses revealed that, in general, participants were increasingly likely to reject across the twelve trials. None of our other analyses revealed significant effects of trial number or trial number by distribution.

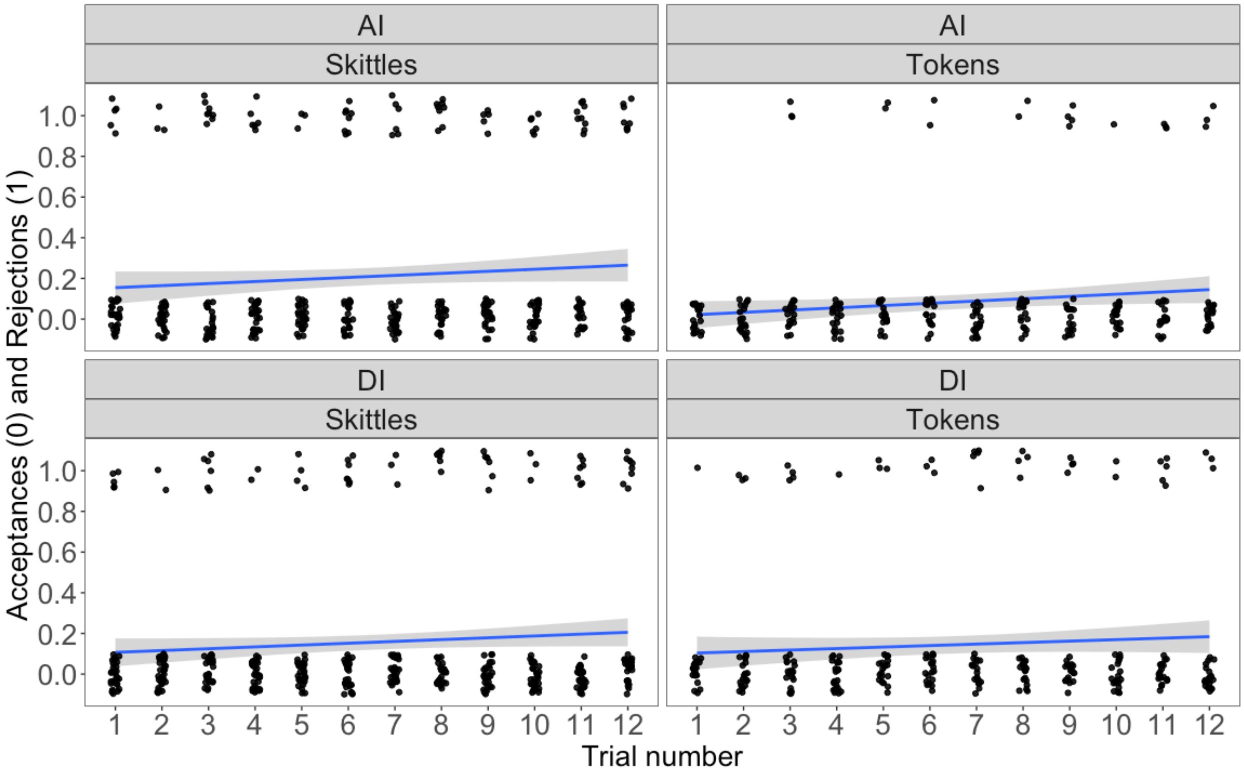

Figure S3. Rejections across trials (trial 1-12). Faceted by condition (AI = advantageous inequity; DI = disadvantageous inequity) and resource type.

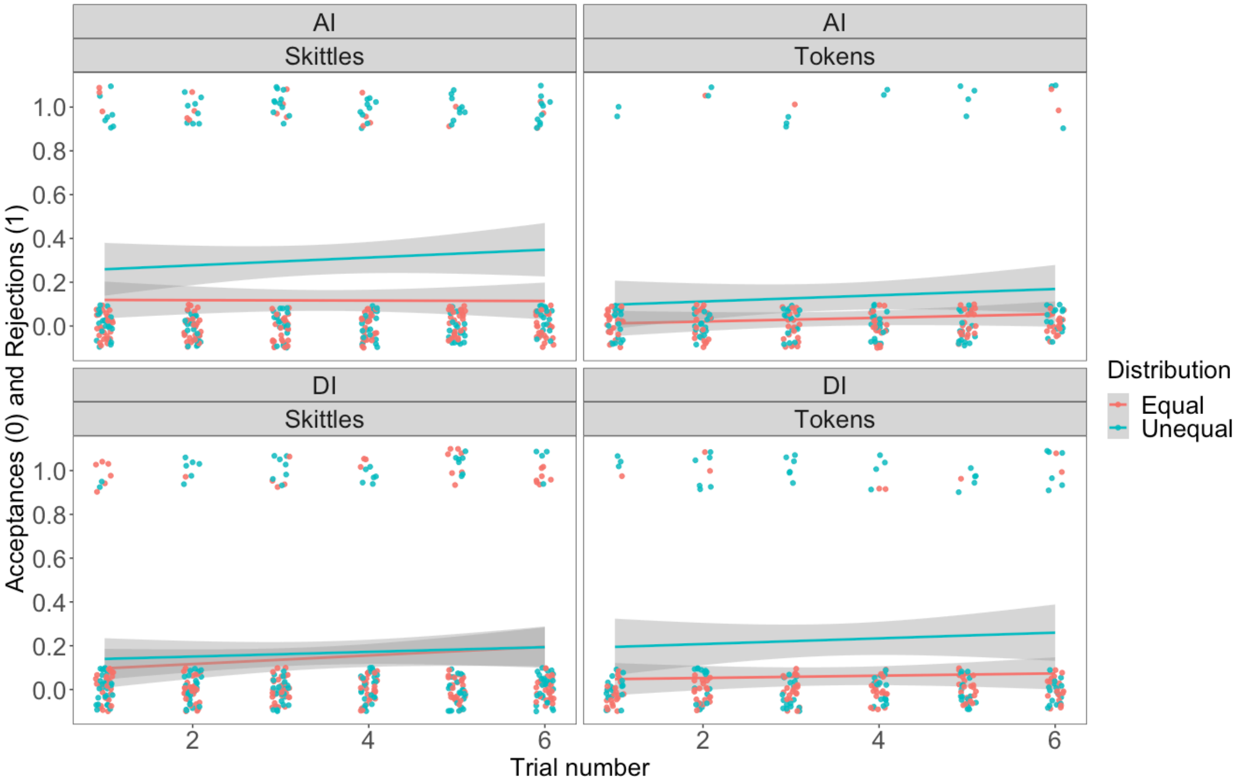

Figure S4. Rejections across trials by distribution (trials 1-6). Faceted by condition (AI = advantageous inequity; DI = disadvantageous inequity) and resource type.

Table S5. Estimates and bootstrapped CIs (N = 1,000 simulations) of fixed effects in Generalized Linear Mixed Model predicting children's rejections (= 1) in the Inequity Game. These models include trial number (1-12; three models on the left) or trial by distribution (1-6; three models on the right). Full models include a term for condition (DI = disadvantageous inequity; AI = advantageous inequity). Baselines were set as follow: Condition: AI; Distribution = Equal; Resource type = Skittles. Table also shows goodness-of-fit statistics.

	**Full**	**DI**	**AI**	**Full**	**DI**	**AI**
(Intercept)	-4.11*	-3.03*	-4.39*	-4.20*	-3.20*	-4.44*
	[-4.28; -3.08]	[-3.95; -2.48]	[-9.31; -3.06]	[-5.67; -2.91]	[-4.89; -2.02]	[-10.50; -2.80]
Condition: DI	0.89			0.94		
	[-0.50; 1.54]			[-0.68; 2.38]		
Distribution: Unequal	1.81*	0.12	1.91*	1.95*	0.46	1.67
	[1.17; 2.25]	[-0.58; 0.54]	[1.11; 3.07]	[1.38; 2.64]	[-0.15; 1.21]	[-0.83; 4.15]
Resource Type: Tokens	-1.56*	-1.71*	-1.96	-1.54*	-1.69*	-1.65
	[-3.59; -1.57]	[-2.95; -1.12]	[-9.24; 0.44]	[-3.75; -0.43]	[-3.40; -0.67]	[-7.43; 0.83]
Trial number (1-12)	0.07*	0.07	0.06			
	[0.04; 0.10]	[-0.02; 0.13]	[-0.04; 0.17]			
Condition x Distribution	-1.68*			-1.75*		
	[-2.17; -1.14]			[-2.66; -1.07]		
Condition x Resource type	-0.36			-0.36		
	[-1.17; 1.20]			[-1.91; 2.10]		
Distribution x Resource type	0.04	1.90*	0.04	0.02	1.88*	-0.01
	[-0.70; 1.33]	[0.53; 2.73]	[-1.57; 6.00]	[-0.88; 1.96]	[1.04; 2.20]	[-0.91; 6.09]
Condition x Distribution x Resource type	1.94*			1.94		
	[0.02; 3.00]			[-0.19; 3.62]		
Trial by distribution (1-6)				0.13	0.18	0.06
				[-0.09; 0.30]	[-0.02; 0.47]	[-0.41; 0.43]
Distribution x Trial by distribution				-0.00	-0.07	0.11
				[-0.12; 0.21]	[-0.22; 0.13]	[-0.44; 0.95]
AIC	838.23	472.93	366.57	840.87	475.41	368.03
BIC	889.51	499.77	392.93	897.27	506.72	398.78
Log Likelihood	-409.11	-230.46	-177.28	-409.43	-230.70	-177.01
Number of trials	1246	648	598	1246	648	598
Number of participants	104	54	50	104	54	50
Variance: ID (Intercept)	5.24	3.40	8.57	5.26	3.35	8.77

* 0 outside the confidence interval
