# Supplementary material for: Reward type influences adults’ rejections of inequality in a task designed for children
Source: PLoS One. 2022 Aug 16;17(8):e0272710. doi: 10.1371/journal.pone.0272710 (PMC9380955; doi:10.1371/journal.pone.0272710)
Supplement: Attachment — Submitted filename: Second review.pdf [file pone.0272710.s009.pdf]

I think authors have addressed properly all questions. Two minor caveats (for further work):

* Sorry for the confusion with blocks/allocations. However, as this implies that there is a trial order effect (that authors now consider in the Sup. Mat.), I suggest that for further research authors *use* the block design (E + Unequal), randomizing block order instead of all decisions. I think this could help in the reduction of contamination between trials.

* However, defining correctly (I hope) the equal/unequal decisions, and running a simple regression, design variables can explain differences in rejection rates (Unequal- Equal). Gender variables now become irrelevant:

```
gen t1decE= t1dec if t1dist=="e"
gen t2decE= t2dec if t2dist=="e"
gen t3decE= t3dec if t3dist=="e"
gen t4decE= t4dec if t4dist=="e"
gen t5decE= t5dec if t5dist=="e"
gen t6decE= t6dec if t6dist=="e"
gen t7decE= t7dec if t7dist=="e"
gen t8decE= t8dec if t8dist=="e"
gen t9decE= t9dec if t9dist=="e"
gen t10decE= t10dec if t10dist=="e"
gen t11decE= t11dec if t11dist=="e"
gen t12decE= t12dec if t12dist=="e"

recode t1decE t2decE t3decE t4decE t5decE t6decE t7decE t8decE t9decE t10decE t11decE
t12decE (missing = 0)

gen equal = t1decE+ t2decE+ t3decE+t4decE+t5decE+t6decE+t7decE+t8decE+t9decE+
t10decE+ t11decE+ t12decE
gen unequal = t1dec+ t2dec+ t3dec+t4dec+t5dec+t6dec+t7dec+t8dec+t9dec+ t10dec+
t11dec+ t12dec_paper – equal

regress dif actorage res own_ad resXowm female other_female  same_gender econ
other_econ

      Source |       SS           df       MS      Number of obs   =       102
-------------+----------------------------------   F(9, 92)        =      1.26
       Model |  1.01653388         9  .112948209   Prob > F        =    0.2705
    Residual |  8.26015451        92  .089784288   R-squared       =    0.1096
-------------+----------------------------------   Adj R-squared   =    0.0225
       Total |  9.27668839       101    .0918484   Root MSE        =    .29964

------------------------------------------------------------------------------
         dif |      Coef.   Std. Err.      t    P>|t|     [95% Conf. Interval]
-------------+----------------------------------------------------------------
    actorage |   .0010817   .0026372     0.41   0.683     -.004156    .0063194
         res |   .1847137   .0912823     2.02   0.046     .0034191    .3660083
      own_ad |   .1844242   .0860322     2.14   0.035     .0135569    .3552916
     resXowm |  -.2818712   .1282748    -2.20   0.031     -.536636   -.0271065
      female |   .0879401   .0679329     1.29   0.199    -.0469806    .2228608
other_female |   .0425448   .0670059     0.63   0.527    -.0905347    .1756242
 same_gender |   .0374303    .066879     0.56   0.577    -.0953972    .1702577
        econ |  -.0407902   .0607982    -0.67   0.504    -.1615407    .0799603
  other_econ |   .0724631   .0645547     1.12   0.265    -.0557481    .2006743
       _cons |  -.1374377   .1312655    -1.05   0.298    -.3981422    .1232668
------------------------------------------------------------------------------
```

(still Stata, but I hope the code is easy to follow)
