# Supplementary material for: Reward type influences adults’ rejections of inequality in a task designed for children
Source: PLoS One. 2022 Aug 16;17(8):e0272710. doi: 10.1371/journal.pone.0272710 (PMC9380955; doi:10.1371/journal.pone.0272710)
Supplement: Attachment — Submitted filename: Review PONE-D-21-21504.docx [file pone.0272710.s007.docx]

The paper run an experiment on the Inequity Game that authors have used on previous studies with children to study their social preferences. By design, this not properly a game as there is no strategic interactions and, consequently beliefs are irrelevant in choices. The Nash predictions (“Accept all”) coincide with the efficient solution. Rejections on the second block of the experiment where shares are unequal can be interpreted as a rejection of inequality while rejections of the first part are harder to interpret (rejection of the game? Misunderstanding it?).

The experiment was properly run using a naturalistic setting with the aim of making the results comparable with children’ ones. I think the paper is publishable as it is, but I have some comments (musings) about it. Except the last one, answering them require further data and/or design changes, that is, another paper. I think that for the current study will be enough to address them in the discussion section.

For all the analyses presented below I use the provided data but using Stata instead of R. At the end of the report I have include the code. I hope it is easily translatable into R.

1. The paper weaker point is the lack of enough explanations for differences in behaviours: the two design variables have small effects; the same happens with the demographic variables collected. Maybe some additional tests or experiments could help (big five, etc).
2. In relation with the previous comment, I don’t see any problem in having an unbalanced sample, but as there are few observations per treatment, the power of the study should be also very low, and consequently the explanatory power of the variables.
3. One intriguing result of the paper is the very low rejection rate under most conditions. I can speculate that this low value can be caused by a mix of the following reasons:

* The recruitment system, although more natural than the standard economic lab experiment, can select more collaborative subjects than the general population.

* Also, as the actor and recipient are facing each other, decisions that harm the other are harder to make. In fact, some kind of (at least non-verbal) communication is expected.

* Connected with the previous point, as a third player of the game (the experimenter) takes the distribution decision, rejecting unequal splits can be viewed as an unfair punishment of an innocent player (in contrast with Dawes et al where allocations were random).

* Finally, for adults, the stakes used on the experiment are very low for adults (36 candies or 3.6 $ to share between the two players), this experiment is closer to a hypothetical one than to a payed economic experiment. Very low-stakes make much cheaper the inclination to reject advantageous inequality allocations.

Although the data do not allow addressing the previous points some discussion about them could be worthwhile. Additionally, as authors are interested in comparing children vs. adult behaviours, it is worth discussing how comparable are the stakes among people of different ages: while 2 $ or 20 candies can be attractive for children, soma adults can consider those amounts, especially with monetary rewards, even insulting, so reactions cannot be comparable even if the same experimental design is used.

4. Even as the recipient was passive in the experiment, if the actor has visual contact (as I assumed above) with him/her, observable characteristics can influence actions… More on this on the next point.
5. I’m not familiarized with the GLMM used in the paper to explain the successive accept/reject decisions of subjects. But I think it makes more sense to recodify those into three variables (see the code below): rejection rate for equal distributions, rejection rate for unequal distributions and the difference between these two so that positive values mean higher rejections rates under inequality. A simple t-test show that the rejection rate is higher on the second block.

In this case, the correlation between decisions in the two blocks is clearly observed, so it make sense to use decisions on the first block to explain behaviour on the second one. The fixed-effect model used on the paper partially capture this idea.

As the first graphs show nor only rejections depend on the interaction between design variables but also the increase in rejections. The gender of the actor seems to matter (but remember the point about power), but more relevantly, interactions between players of the same gender seem to be very different to mixed-gendered couples who don’t increase their rejections at all.

For the econometric tests I run Tobit regressions as the rejection rates are bounded, but maybe hurdle models will make more sense in this context as rejections are heavily skewed toward 0.

In the rejection rate of the first block, the only relevant demographic variable is age (but the sample age is skewed). More problematic is the significant interaction between design variables. It is sensible that resource type can influence behaviour (but remember that there are no motives to reject assignments in this block) but the significant interaction indicates that there are behavioural spillovers between blocks. Authors should have used order randomization to reduce those (or a surprise restart, so that subjects do not know about their position on the second block when making their decisions).

The last two models, sadly confirm that design variables only influence through the first block decisions: ie. Conditions affect behaviour since they are known. But, the gender pairing is very relevant. I think authors have to expand the results section to consider this last point. Of course, it will be very interesting to know if this effect also appear in children experiments (even adolescents, glups!).

**STATA CODE** (I also include the graphs and regressions outputs)

Variable definitions

-------------------

gen equal = t1dec+ t2dec+ t3dec+ t4dec+ t5dec+ t6dec

gen unequal = t7dec+ t8dec+ t9dec+ t10dec+ t11dec+ t12dec_paper

gen rej_rate_equal = 1-equal/6

gen rej_rate_unequal = 1-unequal/6

gen dif = rej_rate_unequal- rej_rate_equal

// The following are transformations into binary variables for the regressions

gen res = resource=="Tokens"

gen own_ad = condition=="AI"

gen resXowm= res* own_ad

gen female = actorgender=="F"

gen other_female = recipientgender=="F"

gen same_gender = 1 if female==other_female

replace same_gender = 0 if same_gender==.

gen equal_gender = "Same gender" if same_gender==1

replace equal_gender = "Different gender" if same_gender==0

gen econ = actorecon=="Y"

gen other_econ= recipientecon=="Y"

Graphs

-----

graph bar (mean) dif , over(condition) over(resource)

graph bar (mean) dif , over( actorgender) over(condition) over(resource)

graph bar (mean) dif , over(condition) over(resource) over( equal_gender )

```
Statistics
----------
ttest rej_rate_equal == rej_rate_unequal
corr rej_rate_unequal rej_rate_equal res own_ad  resXowm


Models
------
tobit rej_rate_equal actorage  res own_ad resXowm female other_female same_gender econ other_econ , ll(0) ul(1)

Tobit regression                                Number of obs     =        103
                                                   Uncensored     =         38
Limits: lower = 0                                  Left-censored  =         64
        upper = 1                                  Right-censored =          1

                                                LR chi2(9)        =      19.15
                                                Prob > chi2       =     0.0239
Log likelihood = -53.094854                     Pseudo R2         =     0.1528

---------------------------------------------------------------------------------------
       rej_rate_equal |      Coef.   Std. Err.      t    P>|t|     [95% Conf. Interval]
----------------------+----------------------------------------------------------------
             actorage |   .0109479   .0039331     2.78   0.007     .0031387    .0187571
                  res |  -.0033594   .1396663    -0.02   0.981    -.2806702    .2739513
               own_ad |     .15165   .1256607     1.21   0.231    -.0978522    .4011523
              resXowm |  -.4262327   .2105828    -2.02   0.046    -.8443499   -.0081156
               female |   .0142697   .1057135     0.13   0.893    -.1956269    .2241663
         other_female |  -.0166856   .1053692    -0.16   0.875    -.2258987    .1925275
          same_gender |   .0218872   .1050358     0.21   0.835    -.1866637    .2304382
                 econ |   .1103916   .0936026     1.18   0.241    -.0754585    .2962417
           other_econ |   .1281356   .1024566     1.25   0.214    -.0752943    .3315655
                _cons |  -.5133934   .2060973    -2.49   0.014    -.9226044   -.1041825
----------------------+----------------------------------------------------------------
 var(e.rej_rate_equal)|   .1514945    .040397                      .0892192    .2572382
---------------------------------------------------------------------------------------

tobit rej_rate_unequal actorage rej_rate_equal res own_ad resXowm female other_female same_gender econ other_econ , ll(0) ul(1)

Tobit regression                                Number of obs     =        102
                                                   Uncensored     =         45
Limits: lower = 0                                  Left-censored  =         56
        upper = 1                                  Right-censored =          1

                                                LR chi2(10)       =      81.81
                                                Prob > chi2       =     0.0000
Log likelihood = -28.277848                     Pseudo R2         =     0.5912

-----------------------------------------------------------------------------------------
       rej_rate_unequal |      Coef.   Std. Err.      t    P>|t|     [95% Conf. Interval]
------------------------+----------------------------------------------------------------
               actorage |  -.0013527    .003018    -0.45   0.655    -.0073468    .0046413
         rej_rate_equal |   1.603277   .1988036     8.06   0.000     1.208436    1.998118
                    res |  -.0304914   .0953644    -0.32   0.750    -.2198932    .1589104
                 own_ad |   .0069207   .0877303     0.08   0.937    -.1673191    .1811606
                resXowm |   .0363785   .1370126     0.27   0.791    -.2357404    .3084974
                 female |  -.1108585   .0752535    -1.47   0.144    -.2603185    .0386016
           other_female |   .1223106   .0732215     1.67   0.098    -.0231136    .2677348
            same_gender |   .2114181   .0748137     2.83   0.006     .0628316    .3600047
                   econ |   -.023042   .0643301    -0.36   0.721     -.150807    .1047231
             other_econ |   .0781438    .068026     1.15   0.254    -.0569618    .2132494
                  _cons |  -.3083541   .1340263    -2.30   0.024    -.5745418   -.0421663
------------------------+----------------------------------------------------------------
 var(e.rej_rate_unequal)|   .0667926   .0155187                       .042104    .1059581
-----------------------------------------------------------------------------------------


regress dif actorage res own_ad resXowm female other_female  same_gender econ other_econ


      Source |       SS           df       MS      Number of obs   =       102
-------------+----------------------------------   F(9, 92)        =      1.61
       Model |  .352479548         9  .039164394   Prob > F        =    0.1234
    Residual |  2.23575576        92  .024301693   R-squared       =    0.1362
-------------+----------------------------------   Adj R-squared   =    0.0517
       Total |  2.58823531       101  .025626092   Root MSE        =    .15589

------------------------------------------------------------------------------
         dif |      Coef.   Std. Err.      t    P>|t|     [95% Conf. Interval]
-------------+----------------------------------------------------------------
    actorage |   .0002481    .001372     0.18   0.857    -.0024768    .0029731
         res |   .0217637   .0474903     0.46   0.648    -.0725561    .1160835
      own_ad |   .0451294   .0447589     1.01   0.316    -.0437656    .1340243
     resXowm |  -.0665722   .0667359    -1.00   0.321    -.1991154     .065971
      female |  -.0457704   .0353426    -1.30   0.199    -.1159638    .0244231
other_female |   .0452063   .0348603     1.30   0.198    -.0240292    .1144419
 same_gender |   .1115898   .0347943     3.21   0.002     .0424853    .1806943
        econ |  -.0023715   .0316307    -0.07   0.940    -.0651928    .0604498
  other_econ |   .0446137    .033585     1.33   0.187    -.0220891    .1113165
       _cons |  -.0582882   .0682918    -0.85   0.396    -.1939216    .0773452
------------------------------------------------------------------------------
```
