# Supplementary material for: Reward type influences adults’ rejections of inequality in a task designed for children
Source: PLoS One. 2022 Aug 16;17(8):e0272710. doi: 10.1371/journal.pone.0272710 (PMC9380955; doi:10.1371/journal.pone.0272710)
Supplement: Attachment — Submitted filename: Reward type_R1_Response to reviewers.docx [file pone.0272710.s008.docx]

**Journal Requirements**

When submitting your revision, we need you to address these additional requirements.

1. Please ensure that your manuscript meets PLOS ONE's style requirements, including those for file naming. The PLOS ONE style templates can be found at

<https://journals.plos.org/plosone/s/file?id=wjVg/PLOSOne_formatting_sample_main_body.pdf> and

<https://journals.plos.org/plosone/s/file?id=ba62/PLOSOne_formatting_sample_title_authors_affiliations.pdf>

**We have done this.**

2. Please provide additional details regarding participant consent. In the Methods section, please ensure that you have specified (1) whether consent was informed and (2) what type you obtained (for instance, written or verbal). If your study included minors, state whether you obtained consent from parents or guardians. If the need for consent was waived by the ethics committee, please include this information.

**We have added a section entitled “Ethical note” to our method that details the Harvard IRB protocol number as well as that we obtained written informed consent.**

3. Please include captions for your Supporting Information files at the end of your manuscript, and update any in-text citations to match accordingly. Please see our Supporting Information guidelines for more information: <http://journals.plos.org/plosone/s/supporting-information>.

**We have done this.**

4. While revising your submission, please upload your figure files to the Preflight Analysis and Conversion Engine (PACE) digital diagnostic tool, <https://pacev2.apexcovantage.com/>. PACE helps ensure that figures meet PLOS requirements. To use PACE, you must first register as a user. Registration is free. Then, login and navigate to the UPLOAD tab, where you will find detailed instructions on how to use the tool. If you encounter any issues or have any questions when using PACE, please email PLOS at figures@plos.org. Please note that Supporting Information files do not need this step.

**We have done this.**

**REVIEWER 1**

Comment R1.1

The paper run an experiment on the Inequity Game that authors have used on previous studies with children to study their social preferences. By design, this not properly a game as there is no strategic interactions and, consequently beliefs are irrelevant in choices. The Nash predictions (“Accept all”) coincide with the efficient solution. Rejections on the second block of the experiment where shares are unequal can be interpreted as a rejection of inequality while rejections of the first part are harder to interpret (rejection of the game? Misunderstanding it?).

The experiment was properly run using a naturalistic setting with the aim of making the results comparable with children’ ones. I think the paper is publishable as it is, but I have some comments (musings) about it. Except the last one, answering them require further data and/or design changes, that is, another paper. I think that for the current study will be enough to address them in the discussion section.

For all the analyses presented below I use the provided data but using Stata instead of R. At the end of the report I have include the code. I hope it is easily translatable into R.

**Response R1.1.**

**Our thanks to Reviewer 1 for their thoughts on our paper and their assessment that the paper is publishable as is.**

**It was really interesting for us to consider these new perspectives on our work, and we have made several additions to our manuscript. In line with this overview comment, our approach in our revision has been to address as much as possible in the discussion and, where possible, in the methods and analyses. For other comments, we have noted that we will consider them in future lines of work stemming from this study and remain grateful to the reviewer for their suggestions. Additionally, we are grateful to the reviewer for engaging as much as they did with our data and for providing new graphs and code.**

Comment R1.2.

**1.** The paper weaker point is the lack of enough explanations for differences in behaviours: the two design variables have small effects; the same happens with the demographic variables collected. Maybe some additional tests or experiments could help (big five, etc).

**Response R1.2.**

**We agree that future work would benefit from gathering additional individual-level data to help explain patterns of inequity aversion. Indeed, this is a direction we are currently exploring in our developmental work (and we now mention this on p. 21).**

Comment R1.3.

**2.** In relation with the previous comment, I don’t see any problem in having an unbalanced sample, but as there are few observations per treatment, the power of the study should be also very low, and consequently the explanatory power of the variables.

**Response R.1.3.**

**We have now included the code for our sensitivity analysis in our code file as supplementary material. Apologies for not including our R code in our previous submission. Additionally, we now discuss low power as a limitation in our revised discussion and agree that this aspect of our design was worth highlighting (p. 20).**

**Comment R1.4.**

**3.** One intriguing result of the paper is the very low rejection rate under most conditions. I can speculate that this low value can be caused by a mix of the following reasons:

* The recruitment system, although more natural than the standard economic lab experiment, can select more collaborative subjects than the general population.

* Also, as the actor and recipient are facing each other, decisions that harm the other are harder to make. In fact, some kind of (at least non-verbal) communication is expected.

* Connected with the previous point, as a third player of the game (the experimenter) takes the distribution decision, rejecting unequal splits can be viewed as an unfair punishment of an innocent player (in contrast with Dawes et al where allocations were random).

* Finally, for adults, the stakes used on the experiment are very low for adults (36 candies or 3.6 $ to share between the two players), this experiment is closer to a hypothetical one than to a payed economic experiment. Very low-stakes make much cheaper the inclination to reject advantageous inequality allocations.

Although the data do not allow addressing the previous points some discussion about them could be worthwhile. Additionally, as authors are interested in comparing children vs. adult behaviours, it is worth discussing how comparable are the stakes among people of different ages: while 2 $ or 20 candies can be attractive for children, soma adults can consider those amounts, especially with monetary rewards, even insulting, so reactions cannot be comparable even if the same experimental design is used.

**Response R1.4.**

**We thank the reviewer for raising these points and have endeavored to address each of them in our revised discussion (p. 18-19; p. 20-21). We agree that it is interesting and important to highlight the low levels of rejections across the board, which is an interesting difference from work with children using this same task (see p. 21).**

**Comment R1.5.**

**4.** Even as the recipient was passive in the experiment, if the actor has visual contact (as I assumed above) with him/her, observable characteristics can influence actions… More on this on the next point.

**Response R1.5.**

**Thanks for raising this. We now address this point in our revised discussion (p. 21).**

**Comment R1.6.**

**5.** I’m not familiarized with the GLMM used in the paper to explain the successive accept/reject decisions of subjects. But I think it makes more sense to recodify those into three variables (see the code below): rejection rate for equal distributions, rejection rate for unequal distributions and the difference between these two so that positive values mean higher rejections rates under inequality. A simple t-test show that the rejection rate is higher on the second block.

In this case, the correlation between decisions in the two blocks is clearly observed, so it make sense to use decisions on the first block to explain behaviour on the second one. The fixed-effect model used on the paper partially capture this idea.

As the first graphs show nor only rejections depend on the interaction between design variables but also the increase in rejections. The gender of the actor seems to matter (but remember the point about power), but more relevantly, interactions between players of the same gender seem to be very different to mixed-gendered couples who don’t increase their rejections at all.

For the econometric tests I run Tobit regressions as the rejection rates are bounded, but maybe hurdle models will make more sense in this context as rejections are heavily skewed toward 0.

In the rejection rate of the first block, the only relevant demographic variable is age (but the sample age is skewed). More problematic is the significant interaction between design variables. It is sensible that resource type can influence behaviour (but remember that there are no motives to reject assignments in this block) but the significant interaction indicates that there are behavioural spillovers between blocks. Authors should have used order randomization to reduce those (or a surprise restart, so that subjects do not know about their position on the second block when making their decisions).

The last two models, sadly confirm that design variables only influence through the first block decisions: ie. Conditions affect behaviour since they are known. But, the gender pairing is very relevant. I think authors have to expand the results section to consider this last point. Of course, it will be very interesting to know if this effect also appear in children experiments (even adolescents, glups!).

**Response R1.6.**

**Thanks for thinking through the analysis approach and raising the issue of rejections across trials. The reason we opted for Generalized Linear Mixed Models is because it preserves our binary data in its original form and allows us to use data from all trials (rather than calculating means) while controlling for repeated measures through the inclusions of random intercepts for each participant ID (i.e., to account for the fact that some participants may, in general, be more likely to reject than others). Generalized Linear Mixed Models as implemented in R using the package ‘lme4’ are what have typically been done in studies using the Inequity Game and related paradigms (e.g. House et al., 2013, *PNAS*; Blake et al., 2015, *Nature*). GLMMs are often used in psychology and behavioral ecology more generally (please see Bolker et al., 2009, *Trends in Ecology and Evolution* for an overview).**

**We are very appreciative of the time the reviewer invested in exploring our data and apologize for not including our R code with our original submission to help facilitate the reproduction of our results. We now include our R code in this submission. Our team is not familiar with STATA so this may well be a misunderstanding of how the included code dealt with the data. However, one of the early lines seems to assume that the equal trials were trials 1-6 while the unequal trials were trials 7-12, when in fact, trial distribution was randomized across the 12 trials.**

**Specifically, this section:**

**gen equal = t1dec+ t2dec+ t3dec+ t4dec+ t5dec+ t6dec**

**gen unequal = t7dec+ t8dec+ t9dec+ t10dec+ t11dec+ t12dec_paper**

**If this is the case, the difference score would not be calculated correctly.**

**Regardless, we agree that it is interesting to ask whether behavior varies across time and now include an exploration of this in our SOM (see Figures S3 and S4 along with Table S5), which we now mention on p. 14 in our revised MS (indeed, Reviewer 2 also suggested that we include an analysis of this kind). Where we respectfully disagree, however, is the way in which changes across time should be examined. In the code included with Reviewer 1’s review, block was examined as a unit of interest. We think this is because of a confusion in how we initially described our design which we have now corrected (p. 12). Specifically, in our original design description we erroneously used the term block when in fact trials were randomized. Because distribution (equal and unequal trials) was randomized across the 12 trials, there was no meaningful distinction built into our design between the first 6 trials and the last 6 trials. Consequently, we believe an exploration of decisions across time should be done at the trial level, and we now include a model that includes trial number (1-12) as a predictor. We have additionally included models with trial by distribution (trials 1-6 of equal allocation and trials 1-6 of unequal allocations) to explore whether decisions vary across repeated presentations of a given kind of distribution. We have plotted these effects by condition (advantageous and disadvantageous) and resource types (see Figs. S3 and S4).**

**In our models examining trials by distribution (1-6 of equal and unequal trials), we examine the interaction between trial number (1-6) and distribution (equal and unequal). Finally, we conducted these two categories of trial analysis separately for AI and DI conditions to mirror the analytical approach we present in the main text. As Table S5 shows, these analyses revealed that, in general, participants were increasingly likely to reject across the twelve trials. We now describe this in our revised supplement. None of our other analyses revealed a clear effect of trial number or trial number by distribution. The code for these analyses is included in our revised R code file, which is uploaded as supplementary material.**

**For these supplementary analyses, we chose not to explore gender and age interactions because, as the reviewer suggests, we would almost certainly be underpowered to detect these effects, and they were not part of our original hypotheses. However, we now include a section in our revised discussion (p. 21) suggesting that future work would benefit from more systematically pairing participants in gender-matched or different-gender pairs to explore potential dyadic gender composition effects.**

**REVIEWER 2**

Comment R2.1.

The paper shows an interesting experiment 2x2x2 about inequality, a mini dictator game (iterated), with 104 decision makers conducted in 2013, for its realization they use a contraption, the machine is very ingenious, and we think it adds a plus to the experiment, in which players can participate regardless of their age, despite that the age criteria of this experiment are subjects over 18 years old. The actors make two decisions, (1,1 vs 0,0) and (1,4 or 4,1 vs 0,0) and each decision is made 6 times, all decision was paid, and the individual has direct information about the accumulated amount

**Response R2.1.**

**Thank you for your positive evaluation of our paper and for your helpful comments on how to improve it.**

Comment R2.2.

We would need to read information about statistical power analysis.

**Response R2.2.**

**We now provide code for our sensitivity analysis in our code file which is uploaded as supplementary material. Apologies for not including this in our original submission. Note that we include sensitivity analyses because we did not originally conduct power analyses and instead based our target sample on other work that has used the Inequity Game. Since collecting these data, standard research practices in psychology have evolved and we now more regularly base sample sizes on power analyses.**

Comment R2.3.

In the regression model we would like to see different models in which you add the iterations one by one and increase the number of controls by adding the information you have available about the recipient. We would like to look at the p-values in the regression table and you must add cluster for individuals. You must add the analysis code to see the replication when performing an analysis with random values.

**Response R2.3.**

**We have added our analysis code as supplementary material. We hope that this will help reproduce our results and apologies for not including it in our previous submission. Our modeling approach was to test hypothesis-driven models, an approach that is common in developmental studies, but one that we recognize varies across disciplines. From what we understand, the more common approach in economics is to include a table with variables added sequentially as is suggested here. If the editor and reviewer believe that this is an important addition, we can include a table like this in our supplement.**

**We also opted to report p-values from model comparisons (likelihood ratio tests) rather than p-values associated with particular effects in our model output because the developers of the ‘lme4’ package have cautioned against interpreting p-values from the glmer output as these p-values are not especially reliable (**[**https://bbolker.github.io/mixedmodels-misc/glmmFAQ.html#what-are-the-p-values-listed-by-summaryglmerfit-etc.-are-they-reliable**](https://bbolker.github.io/mixedmodels-misc/glmmFAQ.html#what-are-the-p-values-listed-by-summaryglmerfit-etc.-are-they-reliable)**). In our tables showing model estimates we display bootstrapped confidence intervals and highlight cases in which the interval does not include 0.**

**Finally, we are slightly confused by the note that we must add cluster for individuals (which might be the right approach if we were to use a different modeling approach), as we believe that we have essentially done this by including ‘individual’ as a random effect in our Generalized Linear Mixed Models that allows the inclusion of both fixed and random effects. Specifically, by including participant ID as a random effect, we are controlling for repeated measures at the level of the individual. The variance associated with this random effect term is shown in all model output tables.**

Comment R2.4.

They don’t analyze the consistency in the decisions. Including inconsistent subjects in the analysis implies that we do not have a model that includes rounds decisions. And maybe the study could show if the accumulated amount modifies the result. We would add the analysis of the individual's first decision only, to show the robustness of the model.

**Response R2.4.**

**Thanks for this suggestion. We have now included analyses of decisions over trial in our supplemental material and mention these analyses in our revised MS on p. 14. As we detail in Response R1.6, we examined this question in two ways. Our first approach was to examine whether rejections changed across all twelve trials, and we did this overall and then separately for the DI and AI conditions. Our second approach was to examine whether rejections changed across the 6 trials of each allocation type (equal vs unequal) and we did this, again, first overall and then separately for DI and AI conditions. These analyses revealed that rejections tended to increase across the twelve trials but none of our other analyses revealed a significant effect of trial (see Figures S3 and S4 and Table S5 and please see Response R1.6 for more details). We opted for this approach to addressing the question of whether behavior varied across time as opposed to examining first trial behavior only because allocation type was randomized and thus some participants started with an unequal trial while others started with an equal trial. This resulted in few rejections per cell (see table below) which make us reluctant to conduct and formal analyses on first trials alone.**

**Table 1.** First trial behavior

|  |  |  | Accept | Reject |
| --- | --- | --- | --- | --- |
| Advantageous Inequity | Skittles | Equal | 16 | 3 |
|  |  | Unequal | 9 | 2 |
|  | Tokens | Equal | 12 | 0 |
|  |  | Unequal | 8 | 0 |
| Disadvantageous Inequity | Skittles | Equal | 15 | 4 |
|  |  | Unequal | 12 | 1 |
|  | Tokens | Equal | 11 | 0 |
|  |  | Unequal | 10 | 1 |

Comment R2.5.

We find it interesting if you have the explanations of the individuals when making the changes in their decisions, to analyze that motivation, using a Natural Language tool.

**Response R2.5.**

**We agree that an exploration of participants’ explicit reasons for their behavior would be interesting. However, we are unfortunately not in a strong position to examine such responses for two main reasons. First, it is not clear to us that live coders systematically recorded participants’ explanations verbatim, and when an attempt was made to transcribe responses from video, there was frequent interference from background noise (because the study was being run in a rather busy outdoor environment). Second, the most interesting responses would be answers to the question of why people rejected distributions; we could then explore whether explanations varied by condition (AI vs DI) and resource type. However, because rejections were rather rare in general (as we now discuss on P. 21 of our revised MS), our cell sizes for explanations in each category are small, greatly constraining the strength of any inferences that could be drawn. We have now added a section to our revised discussion (p. 21) noting that future studies of this kind should endeavor to more systematically record participant’s explicit reasons and that systematic analysis, making use of natural language tools, could be used to analyze responses.**

Comment. R2.6.

Regarding the comparison of the children's data, the data are not available in the additional information you provide so we do not have enough information to review that analysis. we would like to see a more aggregate behavior of the children's data compared to an aggregate of the adult data.

**Response R2.6.**

**We now include the data from the child study as part of our supplemental materials. Due to differences between the studies’ respective designs, our preference is to keep these analyses at the qualitative level as more of a cursory look at how behavior in our present study compares to previous data. We are concerned that providing a more quantitative approach would create an impression that the study designs were more similar than they were. We hope that by including these data, we open the door for others’ explorations of these effects. If you and the editor feel that a suitably qualified set of analyses would benefit the manuscript, we can certainly include one.**
