# Supplementary material for: Reward type influences adults’ rejections of inequality in a task designed for children
Source: PLoS One. 2022 Aug 16;17(8):e0272710. doi: 10.1371/journal.pone.0272710 (PMC9380955; doi:10.1371/journal.pone.0272710)
Supplement: Attachment — Submitted filename: Response regarding IRB docs.docx [file pone.0272710.s010.docx]

We have now included documents relating to our IRB approval. The two docs relevant to this project are the amendment approvals: One shows approval for working with adults. The other shows approval for asking participants if they have taken a university-level economics course. These amendments were made to the Warneken lab's main protocol (F18470): "The developmental origins of human cooperation". In case it's relevant, we have also included the text for the main protocol.
